# Multicolour nanoscopy of fixed and living cells with a single STED beam and hyperspectral detection

**DOI:** 10.1038/srep46492

**Published:** 2017-04-18

**Authors:** Franziska R. Winter, Maria Loidolt, Volker Westphal, Alexey N. Butkevich, Carola Gregor, Steffen J. Sahl, Stefan W. Hell

**Affiliations:** 1 Department of NanoBiophotonics, Max Planck Institute for Biophysical Chemistry, Am Fassberg 11, 37077 Göttingen, Germany

## Abstract

The extension of fluorescence nanoscopy to larger numbers of molecular species concurrently visualized by distinct markers is of great importance for advanced biological applications. To date, up to four markers had been distinguished in STED experiments featuring comparatively elaborate imaging schemes and optical setups, and exploiting various properties of the fluorophores. Here we present a simple yet versatile STED design for multicolour imaging below the diffraction limit. A hyperspectral detection arrangement (hyperSTED) collects the fluorescence in four spectral channels, allowing the separation of four markers with only one excitation wavelength and a single STED beam. Unmixing of the different marker signals based on the simultaneous readout of all channels is performed with a non-negative matrix factorization algorithm. We illustrate the approach showing four-colour nanoscopy of fixed and living cellular samples.

Emerging fluorescence nanoscopy or super-resolution microscopy^1^,^2^ methods such as the method called STED^3^,^4^,^5^ are well suited for the study of biological processes, which are often the interplay of various proteins, their higher-order assemblies, as well as other molecular entities (e.g. refs 6, 7). To unravel their details, such as the constituents of a molecular complex, it is essential to observe and discriminate the individual molecular components simultaneously and at nanoscale resolution. Such discrimination of the different markers can in principle be achieved based on the fluorophores’ excitation, emission, lifetime or photostability, i.e., any robustly observable property, or any combination thereof. The need for separation of more markers is, however, accompanied by a rise in complexity of the optical setups, often at the expense of usability and stability.

Moreover, the utilization of several different markers within the same sample results in raw data where the detected fluorescence, often in more than one spectral channel, is a superposition of the emission spectra of the individual markers present. A post-processing algorithm is therefore required to reliably separate the fluorescence signals from the different markers. The most widely employed approach for spectral marker separation is linear unmixing^8^. A pixel-based analysis, linear unmixing assumes pre-determined, fixed reference spectra of the markers, which can be a potential source of artefacts if variations in instrument detection efficiencies are not fully accounted for. Another method is spectral phasor analysis (SPA)^9^, which uses the real and imaginary parts of the Fourier-transformed spectra of individual pixels to create phasor scatter plots. For this method, no knowledge of the reference spectra is required, but demonstrations of SPA to date have been limited to three fluorescent markers^9^. In multispectral confocal imaging of fluorescent microspheres, an algorithm of multivariate curve resolution (MCR) based on an alternating least-squares regression and principal component analysis has been successfully applied^10^,^11^, but such a classification-based algorithm fails when it comes to colocalization. A promising strategy to the general task of inferring the distribution of multiple marker fluorophores with partial spectral overlap is non-negative matrix factorization (NNMF)^12^,^13^,^14^. This method, which we explored in this work on multicolor STED, does not in principle require a priori knowledge of any reference spectra and uses the whole image to calculate the contributions of the individual markers to the detected fluorescence signals. It explicitly takes into account that different marker species are excited at different strength at different wavelengths^12^,^13^,^14^.

To date, a wide range of applications with two-colour separation using various super-resolution methods has been described for fixed and for living specimens. However, as the number of markers increases and exceeds two, the situation changes. To our knowledge, no live-cell super-resolution experiments with three or more separated species have been reported. Even when considering fixed cellular specimens, only a handful of reports were found involving four or more super-resolved fluorescent markers, realizing the nanoscopy methods called GSDIM^15^, STORM^16^,^17^, STED^18^ and PAINT^19^. The respective experiments used elaborate imaging schemes and exploited a combination of excitation, emission, lifetime and/or photostability to obtain the separation of the various fluorophores.

Here, we present a point-scanning implementation of “hyperSTED”: a STED setup capable of distinguishing up to four different markers purely based on their emission spectra (Fig. 1). For this, only one excitation wavelength and a single STED beam are needed (Fig. 1a). Together, the two beams provide the resolution increase by the now well-established strategy of controlled fluorescence de-excitation (‘off’-switching) outside the coordinate-giving doughnut zero of the STED light. Reversibly toggling the fluorophores between the fluorescent ‘on’ and the non-fluorescent ‘off’ state by scanning the pair of beams allows sequential readout of ‘on’-state fluorophores/features from within diffraction-unlimited regions^1^.

The assigning of marker identities based on emission spectra is independent of this resolution enhancement and STED operation. We have thus explored hyperspectral detection as a promising avenue for multicolour nanoscopy. It exploits the ratiometric differences in signals collected within multiple spectral detection bands (colour contrast) of selected fluorophores over a range of emission wavelengths. Since – for even up to four colours – just two laser beam lines need to be utilized and kept superposed, our scheme removes a lot of optical complexity, which benefits setup stability.

## Results

**Figure 1:**
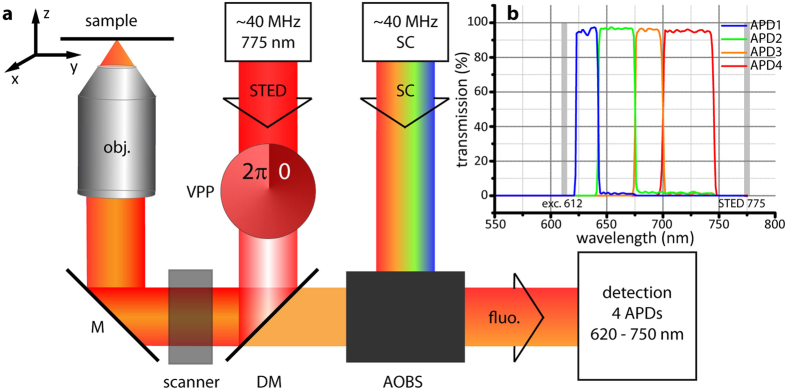
STED nanoscopy with hyperspectral detection (hyperSTED). (**a**) Principle and main components. Different excitation wavelengths (here: up to 8) can be selected from the spectrum of a supercontinuum source (SC) by means of an acousto-optical beam splitter (AOBS). The excitation beam is overlayed with the STED beam at the dichroic mirror (DM). The doughnut-shaped focal STED light distribution for de-excitation is generated by a vortex phase plate (VPP). Both beams pass through a beam scanner and are focused into the sample by the objective (obj.). The fluorescence (fluo.) is recorded by a hyperspectral detection design spanning a total range of 620–750 nm in four channels. M: mirror. A more detailed layout of the optical arrangement is shown in Supplementary Fig. S1 (ref. 20). (**b**) Detection bands of the custom-built hyperspectral detector with avalanche photodiodes 1–4 (APD 1–4). Grey positions indicate wavelengths of excitation and STED de-excitation.

The hyperSTED setup features a hyperspectral detection scheme of four channels (Fig. 1b) covering the range from ~620 nm to ~750 nm, with sharp edges well defined by cascaded filtering (further details in Supplementary Fig. S1). By screening various fluorescent dye pairs with these detection windows in fixed-cell samples, we found that the minimal separation of the emission maxima of two dyes of typical emission spectrum had to be around 20 nm in order for them to be distinguishable after mathematical unmixing of the signals by NNMF (Materials and Methods). Guided by practical experience, we considered and chose to define two dyes distinguishable if the estimated residual crosstalk was <20% when exciting with one excitation wavelength of 612 nm and using a single STED beam at 775 nm for de-excitation.

To present the approach in practice and under realistic bioimaging conditions, a four-colour fixed cell sample was stained with a suitable combination of four of the tested dyes (Table 1): Atto594 for peroxisomes, Abberior Star635P for vimentin, KK1441 for giantin and CF680R for nuclear pores (Materials and Methods). Separation of more than four dyes based on emission alone is not possible with the one excitation wavelength and a set of four detection channels. In this case, the number of channels dictates the maximal number of unique signatures (by relative ratios) that can be accommodated. Here, for example, for four channels and four dyes, the system of the matrix formalism is fully determined (see “Unmixing” in Materials and Methods).

**Figure 2:**
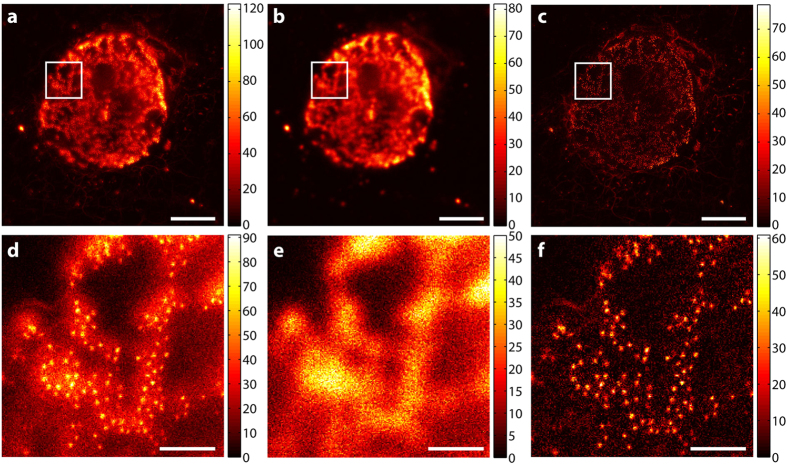
Subtraction of STED-beam contribution to fluorescence excitation. (**a–f**) Data measured in channel 4 (700–750 nm), where the STED-beam excitation (for the dye CF680R) is most severe. (**a**,**d**) Raw data for excitation + STED, (**b**,**e**) raw data for STED-only excitation (separate line step with STED beam active), and (**c**,**f**) data with STED-only excitation subtracted, i.e. “image **a**” – “image **b**”. (**d**–**f**) Enlarged views of corresponding regions in white squares of (**a–c**). Data is counts registered (colourmaps indicated). Scale bars: 5 μm (**a**–**c**), 1 μm (**d**–**f**).

**Figure 3:**
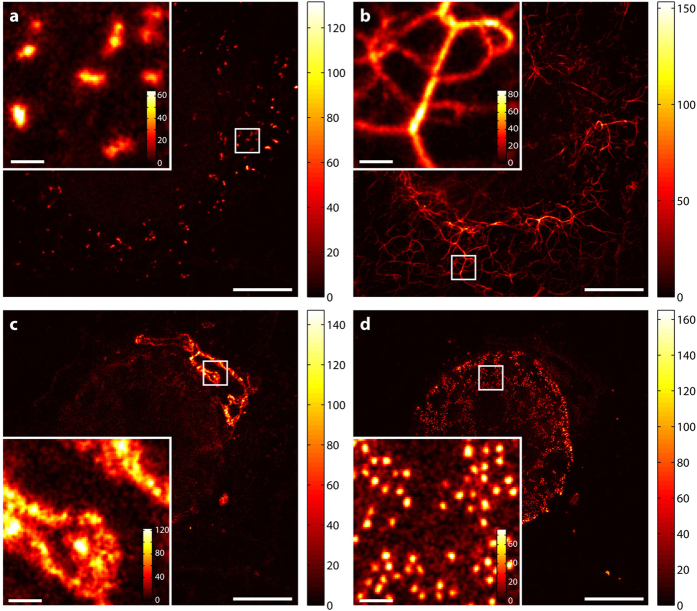
Unmixed and smoothed image data. (**a**) Peroxisomes stained with Atto594. (**b**) Vimentin stained with Abberior Star635P. (**c**) Giantin stained with KK1441. (**d**) Nuclear pores stained with CF680R. Data is counts registered (colourmaps indicated). Smoothing of the image data was performed with a Gaussian of a FWHM of two pixels. Scale bars: 5 μm (whole images), 200 nm (insets).

**Figure 4:**
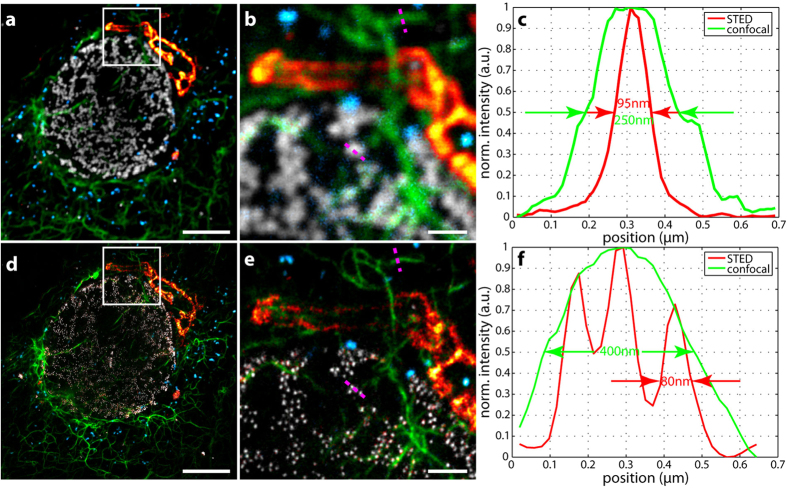
Four-colour hyperSTED data in a fixed cell (unmixed confocal and STED overlays). (**a**) Confocal overlay. (**d**) STED overlay. (**b**,**e**) Enlarged views of regions in white squares, (**b**) confocal and (**e**) STED. (**c**,**f**) Line profiles along the magenta dotted lines indicated in (**b**,**e**) The data of (**e**) was drift corrected such that the maxima of the line profiles coincide. Proteins and fluorescent reporters: blue: peroxisomes – Atto594, green: vimentin – Abberior Star635P, red hot: giantin – KK1441, grey: nuclear pores – CF680R. Scale bars: 5 μm (**a**,**d**), 1 μm (**b**,**e**).

As a prerequisite for good spectral separation performance, each candidate dye has its emission maximum in a distinct and different channel. Each dye can be excited sufficiently well at 612 nm and is efficiently de-excited at 775 nm. Further, as summarized in Table 1, the four chosen dyes for the staining (Figs 2, 3 and 4) have sufficiently different emission spectra such that labelled structures yielded a high image contrast once unmixed (Fig. 3). The applied STED power had to be limited since the CF680R dye exhibits strong excitation by the STED beam alone. For the specific, super-resolved fluorescence signal still to be visible above this induced additional background, the STED power was set to 90 mW. To remove the STED-only excitation background from the data and exclude it in the analysis, its contribution was recorded pixel by pixel in a separate line step. Afterwards it was subtracted from the actual respective channel data (see “Imaging scheme and data analysis” in Materials and Methods). Figure 2 illustrates our observation that the structures recorded in this channel thus became clearer (higher contrast) without losing the high-resolution structural features.

In Figure 3, showing the result of unmixing, the four structures are clearly separated. Some crosstalk and background remain, yet whether this is actual residual crosstalk, remaining excitation from the STED beam that was not fully removed by subtraction, or fluorescence from out-of-focus regions could not be determined. Although still present, the crosstalk is almost not visible with adjusted colourmaps (Supplementary Table S1) that emphasize structures over their ranges of counts useful for interpretation. The estimated bleedthrough of CF680R into the KK1441 channel amounted to around 21% and was the highest, along with the bleedthrough of Atto594 into the Star635P channel (20%) and of Star635P in to the KK1441 channel (20%) and KK1441 into the CF680R channel (19%). All other contributions from dyes to the “wrong” channels were less than 20% (see Table 2). The contributions of up to ~20% may appear rather high given that the separation and overlay can be judged qualitatively to be very good. The numerical result may be influenced by the brightness differences of the different structures. Pixels below a relatively high background threshold of bright structures contribute to the residual crosstalk calculation of dimmer features, leading to higher crosstalk values (see “Residual crosstalk calculation” in Materials and Methods). The value of 20% therefore certainly represents a quite conservative upper limit. To better determine the residual crosstalks, a technical sample with entirely non-overlapping structures and the dyes in question would be needed, which was not available.

For the overlay of all four channels (Fig. 4), the colourmaps were adjusted for improved visualization, with count minima and maxima stated in Supplementary Table S1. This visualization all at one glance clearly emphasizes the resolution improvement when comparing the confocal (Fig. 4a,b) and the STED images (Fig. 4d,e). This is further illustrated in Fig. 4c,f where examples of line profiles across characteristic features (vimentin, nuclear pores) directly show the resolution increase. The STED data was drift-corrected such that the maxima of the respective line profiles coincided. The drift was estimated to be around 100 nm in the 6-minute acquisition time of the STED image. It took only 45 s to acquire the confocal image prior to STED imaging. During this time, the drift should be negligible.

**Figure 5:**
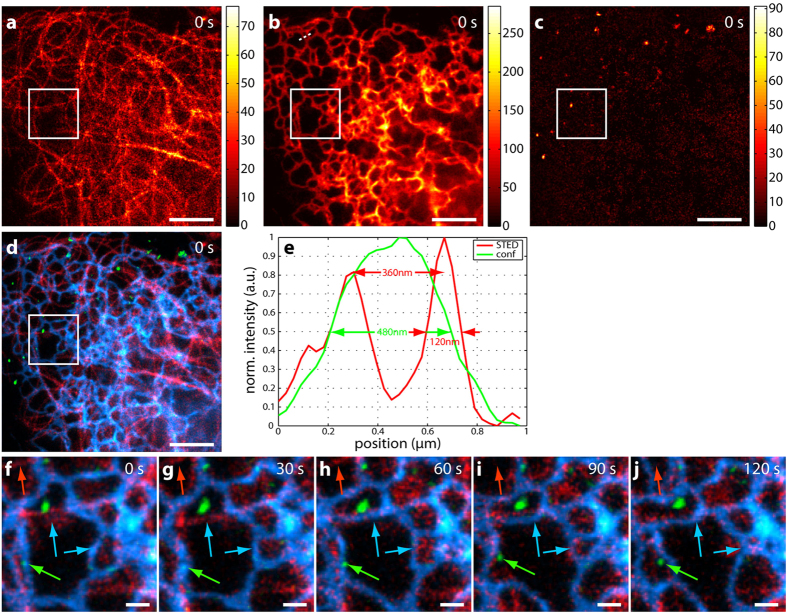
Three-colour live-cell hyperSTED data. U2OS cells were stained with 580CP-docetaxel (tubulin), SiR-Halo-Sec61β (ER) and CF680R-Tat-EGFP (endosomes). (**a**–**c**) Unmixed STED data for 580CP (**a**), SiR (**b**) and CF680R (**c**) of the first of five consecutive frames. Data is counts registered (colourmaps indicated). (**d**) depicts the overlay of (**a**–**c**). Red: 580CP – tubulin, blue: SiR – ER, green: CF680R – endosomes. (**e**) is the line profile along the white dotted line in (**b**). The resolution enhancement due to STED (red line) reveals more structures compared to confocal imaging (green line). (**f**–**j**) Time series of the region marked by the white square. Movements are observed for all structures, i.e. tubulin (red arrow), ER (blue arrows) and endosome (green arrow). Colourmaps are as in (**d**). Scale bars are 3 μm for (**a**–**d**) and 500 nm for (**f**–**j**). Excitation was at 612 nm with 5 μW and a STED power of around 60 mW at 775 nm.

To demonstrate the suitability of the optical setup for multicolour live-cell imaging, a sample of living U2OS cells was prepared (Materials and Methods) and imaged with a power of 5 μW of excitation at 612 nm and a STED power of 60 mW at 775 nm. Figure 5 displays unmixed STED data from a three-colour living cell sample. Tubulin was stained with 580CP-docetaxel derivative (for synthesis see Supplementary Note 1), and the Halo-Sec61β construct, which is expressed in the endoplasmatic reticulum (ER), was labelled with SiR-Halo. Endosomes were visualized via endocytosis of the HIV transactivating protein (Tat peptide fused to EGFP) directly labelled with CF680R (see Supplementary Note 2). Figure 5a–c demonstrates the good separability of the three dyes after unmixing and represents the first of five consecutive frames. The residual crosstalk was determined to be less than 16% (Table 3). Figure 5d shows the overlay of Fig. 5a–c. Tubulin is displayed in red, the ER in blue and endosomes in green. All structures are distinguished by spectrally separating the emission spectra of the attached dyes, even the highly spatially overlapping networks of the ER and tubulin. The use of a common pinhole and the simultaneous acquisition of all four spectral channels result in intrinsically spatially matched raw data sets and therefore unmixed dye channels (Materials and Methods). The resolution improvement with STED becomes clearly visible in Fig. 5e, which contains a comparison of line profiles in STED (red line) and confocal imaging (green line) along the white dotted line in Fig. 5b (ER). With STED, substructures of the ER are discernable. Figure 5f–j depicts the time series of the region marked by the white square. A single frame was acquired in 15 seconds, but the additional recording of the STED-only excitation resulted in a 30-second time difference between two consecutive frames. The coloured arrows highlight movements/structural changes of the associated features (red: tubulin, blue: ER, green: endosomes). Following the red arrow, one notices that the tubulin strand pointed out vanishes in frame 4 (Fig. 5i). The endosome indicated by the green arrow moves to the right and the upper blue arrow points at an ER loop that first appears in frame 3 (Fig. 5h). The lower blue arrow points to the retraction/change of a particular ER loop. For better visualization of the individual structures, the colourmaps were adjusted in Fig. 5d,f–j (minima and maxima stated in Supplementary Table S2).

**Figure 6:**
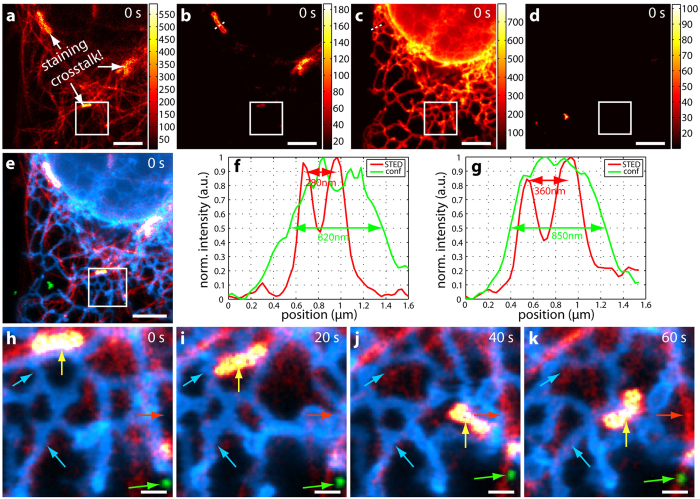
Four-colour live-cell hyperSTED data. U2OS cells were stained with 580CP-docetaxel (tubulin), 620CP-Snap-OMP25 (outer mitochondrial membrane), SiR-Halo-Sec61β (ER) and CF680R-Tat-EGFP (endosomes). (**a**–**d**) Unmixed STED data for 580CP (**a**), 620CP (**b**), SiR (**c**) and CF680R (**d**) of the first of four consecutive frames. Note that only pixels above 10 counts are displayed. Data is counts registered (colourmaps indicated). There is clearly some crosstalk between (**a**) and (**b**) which is due to the staining process and not to the microscope (see text for more details). (**e**) Overlay of (**a**–**d**). Red: 580CP – tubulin, yellow: 620CP – mitochondria, blue: SiR – ER, green: CF680R – endosomes. (**f**,**g**) Line profiles along the white dotted lines in (**b**) and (**c**), respectively. The resolution enhancement due to STED (red lines) becomes clearly visible as the outer mitochondrial membrane is separated (**f**) and a more detailed structure is revealed for the ER (**g**) as compared to confocal imaging (green lines). (**h**–**k**) Time series of the region marked by the white square. Movements of all structures are observed, i.e. tubulin (red arrow), mitochondria (yellow arrow), ER (blue arrows) and endosomes (green arrow). Colourmaps are as in (**e**). Scale bars are 3 μm for (**a**–**e**) and 500 nm for (**h**–**k**). Excitation was at 612 nm with 5 μW and a STED power of around 60 mW at 775 nm.

Next, a four-colour sample of living U2OS cells was prepared and imaged (Materials and Methods), with the unmixed STED data shown in Fig. 6. In addition to the three-colour labelling described above, the fourth dye 620CP-Snap stained the OMP25-Snap construct situated in the outer mitochondrial membrane. Note that Fig. 6a shows significant crosstalk between the mitochondria and tubulin. However, this is not due to the optical setup, since a two-colour sample of 580CP-Halo and 620CP-Snap with Sec61β (ER) and OMP25 (mitochondria) is very well separable (see Supplementary Fig. S3 and Table S5). The reason for this crosstalk could not be determined. It might be due to a crossreaction of 580CP-docetaxel (tubulin) to either the 620CP-Snap construct or the expressed Snap-tag itself, or even unspecific adherence to the mitochondria. Moreover, a similar crosstalk between tubulin and mitochondria was observed when staining a sample with SiR-docetaxel (tubulin) and 620CP-Snap (OMP25, mitochondria). This unwanted crossreaction prevented further useful crosstalk analysis after unmixing. Nevertheless, all structures are clearly distinguishable after unmixing (Fig. 6a–d). Figure 6e displays the overlay of the first of four frames in total where tubulin is shown in red, mitochondria in yellow, the ER in blue and endosomes in green. The resolution enhancement in STED imaging becomes obvious when comparing line profiles between STED (red lines) and confocal data (green lines in Fig. 6f,g). The outer mitochondrial membrane is discernable in STED mode (Fig. 6f) and finer details of the ER are revealed (Fig. 6g). Figure 6h–k contains a time series of the region marked by the white square of four frames in total. Frames are spaced 20 seconds apart, with a STED-only frame recorded in between. Again, coloured arrows show movements/changes of specific features. In the chosen region, one mitochondrion moves around and alters its morphology each frame (yellow arrow). The green arrow points to an endosome that moves upwards and to the left, the red arrow highlights the appearance of a tubulin strand in frame 3 (Fig. 6j) and the blue arrows indicate morphology changes of a specific ER loop (lower blue arrow) or disappearance of one (upper blue arrow). For better visualization of the individual structures, the colourmaps were adjusted in Fig. 6e,h–k, with their minima and maxima stated in Supplementary Table S3.

For the data contained in Figs 5 and 6, the acquisition time was several seconds (see above) for the 12 × 12 μm^2^ areas. This had the advantage that drift as observed in the fixed-cell sample could be neglected. Moreover, the morphology changes clearly indicate that movement and not drifting is observed. Acquisition could be sped up by either omitting the recording of the STED-only excitation when using fluorophores emitting at shorter wavelengths (e.g. 580CP, Atto594, Star635P) or by decreasing the pixel dwell time when the dyes are very bright (e.g. Atto594, 620CP) and increasing the power of the excitation light. It is also possible to acquire data in which the frames are several minutes apart if the dynamic to be observed is slower. The total number of frames that can be taken mainly depends on the photostability of the used fluorophores. Here, the limiting dye was 580CP-docetaxel (tubulin) which exhibited the strongest bleaching (~50% from frame one to five in the three-colour data set), whereas SiR-Halo still generated ~80% of its original counts in frame five.

## Discussion and Conclusions

We have presented hyperSTED, a simple and stable yet versatile STED instrument design for multicolour nanoscopy with only two laser lines. By hyperspectral detection of four channels, separation of four different markers was achieved both in fixed and living cellular samples with one excitation and one STED wavelength. Discrimination of the markers purely based on differences in emission spectra allowed for fast and simultaneous data acquisition.

The experimentally determined crosstalk in a four-colour fixed and a three-colour living cellular nanoscopy application presented no problem for the visualization of the respective structures. In the case of a four-colour living cellular specimen, the individual structures could still be visualized but with a non-negligible crosstalk between tubulin (580CP) and mitochondria (620CP). This limitation was however not related to the hyperSTED setup and approach but due to a not yet identified crossreaction during the cellular staining process. In any case, the crosstalk must be monitored, as it may hamper quantitative co-localization analyses of overlapping features. The amount of crosstalk is a direct function of how much the dye spectra overlap with each other and how well they coincide with the windows of hyperspectral detection. Accommodating more dyes/spectra over the same spectral range comes with closer emission maxima spacing and hence more overlapping spectra. This then entails compromises in crosstalk and fidelity of assignment. Reducing the number of markers will relax the requirements and improve crosstalk performance considerably. For example, when considering a three-colour sample the residual crosstalk can be as low as <2% in the case of three fluorescent beads species (see Supplementary Fig. S2 and Supplementary Table S4) or <16% in the case of the three-colour living cell sample.

The achieved resolution varied for each dye due to different saturation intensities and therefore STED efficiencies. Fluorescence correlation spectroscopy (FCS) measurements^20^ revealed that the resolution for the bluer dyes, such as Atto594, should be around 75 nm (90 nm) and around 40 nm (50 nm) for the redder dyes, such as CF680R, at a STED power of 90 mW (60 mW). The structures shown in Figs 4, 5 and 6 have widths consistent with this level of resolution at the respective STED powers, accounting for convolution of e.g. antibody-decorated filaments and nuclear pores with the effective STED resolution and smoothing of two pixels during the analysis. Data was smoothed for contrast enhancement (see Materials and Methods) compared to raw data. The latter was deliberately not shown as the four spectral channels exhibited strong bleedthroughs for the individual dyes such that only little information could be gained from inspection of the raw data prior to application of the NNMF scheme for separation. While maximal resolution and determination of the effective point-spread functions (PSF) for each dye were not the focus of this multicolour demonstration, direct deconvolution of the unmixed images with the known effective PSFs is a worthwhile direction for further exploration.

The structures and spectra in relation to the detection channels of the dyes used are found in Supplementary Fig. S4 and Supplementary Figs S5 and S6, respectively. We conclude that, for presently available fluorophores, the presented approach is not readily extendable to more than four unless additional wavelengths of excitation and/or STED de-excitation are introduced. For the 612 nm/775 nm STED scheme, addition of other dyes in the bluer regime was limited in practice due to resolution losses, and towards the far-red regime as a result of prohibitive STED-only excitation.

The minimal separation of the emission maxima was approximately 20 nm in order to ensure residual crosstalk of <20%. When this separation was increased at the expense of the number of markers, this value could be as low as <2% in the case of a three-colour fluorescent bead sample. This clearly demonstrates that the choice of markers in relation to the detection windows plays a crucial role. The number of distinguishable markers could be further increased by combining the present hyperSTED setup with other means of discrimination, for example excitation multiplexing^21^, fluorescence lifetime imaging^22^,^23^ or photostability^18^, as previously reported. This is feasible without changes to the hardware, keeping the setup simple and stable.

The included labelling protocol (Materials and Methods) of two conventional antibodies and a green and a red fluorescent protein in combination with the corresponding dye-conjugated nano-boosters for fixed cellular samples yielded high reliability and versatility. The labelling protocol for four-colour living cellular specimen (Materials and Methods) – involving two plasmids, one Halo- and one Snap-tag, together with direct labelling of tubulin via docetaxel and endosomes by Tat-EGFP – still needs some improvement since unwanted crossreactions have been observed. Nevertheless, both protocols create an excellent platform to explore further multicolour labelling strategies.

Evaluating the promise of the hyperSTED approach as presented here, simulations^20^ indicate that six-colour separation of Abberior Star600, Atto594, DyLight633, Abberior Star635P, KK1441 and CF680R should be possible when adding the lifetime information from ratios of signals in two time gates^22^, if the assumed spectra and lifetimes of these dyes are as stated in Table 1. Note that for such a sample only one excitation wavelength of 612 nm and a single STED beam would be required.

## Materials and Methods

### Optical setup

All measurements were performed on a custom-built setup consisting of two laser sources: a supercontinuum source (EXWB-6, NKT Photonics, Birkerød, Denmark) for excitation and a single STED beam (775 nm, Katana08-HP, Onefive, Zürich, Switzerland) for coordinate-targeted de-excitation in a doughnut mode (Fig. 1 and Supplementary Fig. S1). Both were synchronized at a repetition rate of 40 MHz. An acousto-optical beam splitter allowed for selection of up to eight freely chosen excitation wavelengths within the ranges of the supercontinuum source and the acousto-optical tunable filters. These can be used simultaneously or in sequence. This enables the use of a wide range of dyes for various applications. Measurements shown here were performed with only one excitation wavelength of 612 nm at a power of ~5 μW (at the sample) and a STED power of ~90 mW for fixed cells and ~60 mW for living cells (measured in the back focal plane). A quadscanner^22^ was used to scan the beams across the sample. An optical layout with a listing of the components used can be found in Supplementary Fig. S1, which also shows the phasemask strategy for the creation of a STED doughnut in the focal region. The back-propagating sample fluorescence is detected in four spectral channels, with a common pinhole for stable on-axis alignment. Each channel has an avalanche photodiode (APD) as detector. Together, the four channels span a total range of 130 nm. The width and position of the individual spectral channels are defined by commercial long pass filters.

### Imaging scheme and data analysis

The data recording was carried out in two line steps. Each line was scanned twice with a total pixel dwell time of 10 μs for confocal imaging and 70 μs for STED imaging in fixed cells (60 μs for three-colour and 40 μs for four-colour recordings in living cells). Pixels were 20 × 20 nm^2^ (fixed-cell measurements) and 30 × 30 nm^2^ (live-cell measurements). During the first scan, the excitation and the STED beams were switched on; during the second line step, only the STED beam illuminated the sample. The STED-only excitation data from the second line step was smoothed with a Gaussian of a FWHM of three pixels before it was subtracted from the data of the first line step. Any negative counts were set to zero after this subtraction and before the unmixing operation (Fig. 2). Unmixed data was additionally smoothed with a Gaussian of a FWHM of two pixels (Fig. 3) for contrast enhancement.

### Unmixing

Spectral separation of the individual marker fluorophore species was performed with a non-negative matrix factorization (NNMF) algorithm as suggested by Neher *et al*.^12^,^13^. The measured data ***Y*** is approximated by three different matrices ***A**, **X*** and ***Q**. **A*** is the transfer matrix containing the percentage distribution of the various markers in the different detection channels. ***X*** is the marker distribution per pixel and represents the quantity to be known. ***Q*** takes the excitation wavelength into account by weighting data of a specific wavelength with a constant factor according to the excitability of the fluorophore at this wavelength. Note that for only one excitation wavelength, ***Q*** is a column vector. Thus,


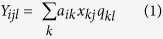


with *a, x, q* the elements of ***A**, **X*** and ***Q***, and where *k* is the index for the number of markers, *i* for the spectral channels, *j* for the pixels and and *l* for the excitation wavelengths. In comparison to Neher *et al*. slight changes to the algorithm’s stability were made and tested in simulations. Initial values of the transfer matrix ***A*** were obtained from single-colour samples. These values were then adjusted manually after each run of the unmixing algorithm for 300 iterations. For each combination of dyes, this had to be repeated around five to fifteen times until the best separation of the dyes was reached. While ***A*** was manually adjusted, the residual crosstalk was calculated after each run of the unmixing algorithm until the off-diagonal elements of the crosstalk matrix *CT* (see Residual crosstalk calculation) were minimized. For this to work best, structures of different shapes (e.g. tubulin, nuclear pores etc.) should be used such that, additionally to *CT*, progress can be judged by eye. However, once ***A*** had been determined for a specific set of dyes, it did not need further adjustment and was used for unmixing of any measurements of that set of dyes. Further, it was found that the best unmixing results were obtained when the manually optimized transfer matrix ***A*** was kept fixed within the unmixing algorithm. Bleaching does not affect the entries of ***A*** as long as the emission spectra do not change by bleaching. The transfer matrices ***A*** for the respective samples are stated in Supplementary Tables S6–S9. The entries of ***Q*** were extracted from the excitation spectra given by the suppliers. 300 iterations were run in each case, in which ***X*** was updated every iteration, and ***Q*** twice in three iterations starting from the last third of total iterations^20^.

### Residual crosstalk calculation

The crosstalk matrix ***CT*** with its elements *ct*_*ki*_ is a quadratic matrix with 100 as its diagonal elements. *CT* needs to be read line-wise, where the off-diagonal elements give the crosstalk value of the investigated dye *k* in the other (wrong) unmixed channels *i*. The values are calculated as follows: After unmixing, a threshold *bg(k*) was set for each unmixed dye *k* such that only the bright pixels 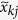 of that dye *x*_*kj*_ remain. Then those pixels *m* were determined where only dye *k* was present. The counts of these pixels were then summed up for each dye individually (*k, r, s, t*) and divided by the sum of the counts of those pixels for the investigated dye *k*, giving the crosstalk matrix elements *ct*_*ki*_.


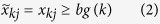



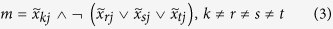



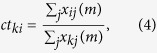


where *k* is the index for the dye, *j* for the pixel and *i* for the channel^20^. 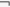, 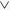, and 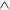 denote the logic ‘not’, ‘or’ and ‘and’ operators, respectively. In case of a three-colour sample, the index *t* is no longer required.

Note that both brightness differences between unmixed channels and the set threshold values *bg(k*) greatly influence the entries of *CT*. Bright channels with a high *bg(k*) give rise to high crosstalk values into dimmer channels with lower thresholds, as pixels below the threshold of the bright channel could be falsely attributed to the dimmer dye. Due to the high brightness difference this might then lead to off-diagonal elements greater than one even when the dyes are well separated. This seems counterintuitive but makes sense since only those pixels are considered where only one dye species is assumed to be present. Hence for best results of residual crosstalk characterization, the threshold values *bg(k*) should be kept as low as possible, unmixed channels should be as equally bright as possible and structures should not be spatially overlapping.

### Sample preparation. Fixed cells

Double transfected Vero cells with vimentin-citrine and Pex3-plum (to label peroxisomes) were fixed with 4% paraformaldehyde (PFA) in phosphate buffered saline (PBS) (137 mM NaCl, 2.68 mM KCl, 10 mM Na_2_HPO_4_, pH 7.4) for 10 min at room temperature. Then Triton X-100 (Sigma Aldrich, München, DE, T8787) in 0.1% PBS (v/v) was added for 5 min at room temperature. Primary antibodies against Nup153 (Abcam, Cambridge, UK, ab24700, 1:100) and giantin (Abcam, Cambridge, UK, ab24586, 1:500) were added and incubated for 1 h at room temperature. The sample was then washed three times for 5 min in PBS with 2% bovine serum albumin (BSA). Secondary antibodies sheep anti-mouse CF680R and goat anti-rabbit KK1441 (Dianova, Hamburg, DE, both 1:50) were added and incubated for 1 h at room temperature. A washing step of three times 5 min in PBS with 2% bovine serum albumin (BSA) was performed. Nanobodies anti-GFP Abberior Star635P and anti-RFP Atto594 (Chromotek, Planegg-Martinsried, DE, gbaAS635p and rba594, both 1:50) were added and incubated for 1 h at room temperature. Then the sample was washed three times 5 min in PBS and mounted in Mowiol with DABCO (25% (w/v) glycerol, 9% (w/v) Mowiol 4–88, 0.1 M Tris/Cl, 0.1% (w/v) 1,4-Diazabicyclo[2.2.2]octane, pH 8.5). The dyes were sourced from the following suppliers: ATTO-TEC GmbH, Siegen, Germany (Atto594); Abberior GmbH, Göttingen, Germany (Abberior Star635P); gift of authors of ref. 24 (KK1441); Biotium Inc., Hayward, US (CF680R).

It was essential to add the nanobodies as the very last step. Otherwise a crossreaction with the primary mouse antibody (Nup153) was observed.

### Living cells

U2OS cells were double-transfected with a plasmid encoding Sec61β and a Halo-tag located in the endoplasmic reticulum (ER) and a plasmid encoding OMP25 and a Snap-tag in the outer mitochondrial membrane (both kindly given by the Rothman Lab, Yale University, New Haven, USA^25^). About 24 h after transfection, the U2OS cells were incubated for 45 min at 37 °C with a silicon rhodamine-Halo derivative^26^ diluted to a final concentration of 500 nM in HDMEM (HEPES-buffered DMEM) and a 620CP^27^-Snap derivative (synthesis see Supplementary Note 1) with a concentration of 375 nM. Afterwards, the cells were washed once in fresh medium and then incubated with 3.2 μM of a 580CP^27^-docetaxel-derivative (Supplementary Note 1) for 45 min at 37 °C in HDMEM supplemented with 10 μM verapamil (Sigma Aldrich, München, DE). Next, 15 μl of a 120 μM solution of a Tat-EGFP construct labelled with CF680R (see Supplementary Note 2) were mixed with 10 μl HDMEM and verapamil on Parafilm (Bemis company Inc., Neenah, US). The coverslip was placed on Parafilm with cells facing down and incubated for 20 min at room temperature. Afterwards, the cells were washed once in fresh medium, then three times with Heparin (Sigma Aldrich, München, DE) diluted to 1 mg/ml in HDMEM supplemented with 10 μM verapamil. Between each washing step, the cells were left in the Heparin solution for 3 min. Finally, the cells were incubated again in fresh medium containing verapamil for 20 min at 37 °C.

Note that, for the three-colour living cell sample, labelling occurred as described above but cells were only transfected with the plasmid encoding Sec61β and a Halo-tag in the ER. Also, the staining with the 620CP-Snap derivative was omitted.

## Additional Information

**How to cite this article**: Winter, F. R. *et al*. Multicolour nanoscopy of fixed and living cells with a single STED beam and hyperspectral detection. *Sci. Rep.* **7**, 46492; doi: 10.1038/srep46492 (2017).

**Publisher's note:** Springer Nature remains neutral with regard to jurisdictional claims in published maps and institutional affiliations.

## Supplementary Material

Supplementary Information

## Figures and Tables

**Table 1:** Fluorescent dyes tested.

Dye	Supplier	Abs. (nm)	Em. (nm)	τ (ns)	Channel
580CP	ref. 27	583	606	3.6 (ref. 27)	APD1
Atto594	ATTO-TEC	601	627	3.2 (ref. 28)	APD1
Abberior Star600	Abberior	604	627	1.2	APD1
620CP	ref. 27	618	646	1.0 (ref. 27)	APD2
DyLight633	Thermo Fischer Scientific	623	649	1.7	APD2
Abberior Star635P	Abberior	633	654	3.0 (ref. 28)	APD2
SiR	ref. 26	650	674	2.7 (ref. 27)	APD3
KK1441	ref. 24 (compound 1a-H)	661	679	2.4 (ref. 24)	APD3
CF680R	Biotium	680	701	1.8 (ref. 28)	APD4

Absorption (Abs.) and emission maxima (Em.) in nm as specified by the supplier, together with experimentally measured lifetimes (τ) in ns and main detection channel, in which the majority of signal is collected.

**Table 2:** Residual crosstalk after unmixing by non-negative matrix factorization (NNMF) for a four-colour fixed-cell sample.

Dye/Channel	Atto594	Abberior Star635P	KK1441	CF680R
Atto594	100	20	15	11
Abberior Star635P	12	100	20	9
KK1441	6	11	100	19
CF680R	8	7	21	100

Percentage crosstalk in % determined in a four-colour fixed-cell sample with Atto594, Abberior Star635P, KK1441 and CR680R as fluorescent markers, when only pixels above 15, 10, 20 and 20 counts were considered for Atto594, Abberior Star635P, KK1441 and CF680R, respectively. The table needs to be read line-wise. The dye whose signal bleeds through is listed left (e.g. read as: 12% of Star635P bleeding into the unmixed Atto594 channel).

**Table 3:** Residual crosstalk after unmixing by non-negative matrix factorization (NNMF) for a three-colour living cell sample.

Dye/Channel	580CP	SiR	CF680R
580CP	100	16	15
SiR	1	100	1
CF680R	6	8	100

Percentage crosstalk in % determined in the first frame of a three-colour living cell sample with 580CP, SiR and CR680R as fluorescent markers, when only pixels above 0.3 counts were considered for all dyes. The table needs to be read line-wise. The dye whose signal bleeds through is listed left (e.g. read as: 16% of 580CP is bleeding into the unmixed SiR channel).
