# Supplementary material for: Multicolour nanoscopy of fixed and living cells with a single STED beam and hyperspectral detection
Source: Sci Rep. 2017 Apr 18;7:46492. doi: 10.1038/srep46492 (PMC5394456; doi:10.1038/srep46492)
Supplement: Supplementary Information [file srep46492-s1.pdf]

Supplementary Information

Multicolour nanoscopy of fixed and living cells with a single STED beam and hyperspectral detection

Franziska R. Winter, Maria Loidolt, Volker Westphal, Alexey N. Butkevich, Carola Gregor, Steffen J. Sahl & Stefan W. Hell

Department of NanoBiophotonics, Max Planck Institute for Biophysical Chemistry, Am Fassberg 11, 37077 Göttingen, Germany

Correspondence should be addressed to S.W.H. (shell@mpibpc.mpg.de) or F.R.W. (fwinter@mpibpc.mpg.de).

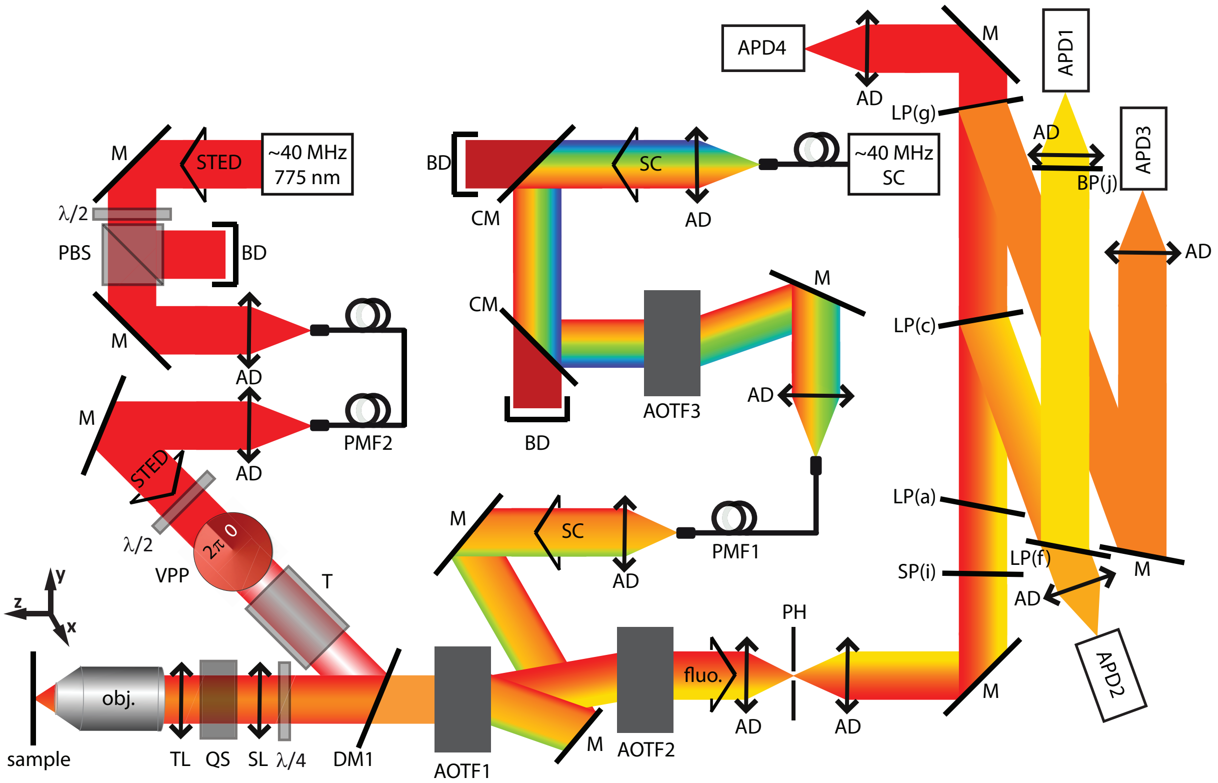

AD	Achromatic doublet	LP	Long pass	PMF	Polarization maintaining fibre
AOTF	Acousto-optical tunable filter	LP(a)	Semrock BLP01-594R-25	QS	Quad scanner
APD	Avalanche photo diode	LP(c)	Semrock BLP01-664R-25	SC	Supercontinuum
BD	Beam dump	LP(f)	Semrock BLP01-633R-25	SL	Scan lens
BP	Band pass	LP(g)	Semrock FF01-692/LP-25	SP	Short Ppass
BP(j)	Semrock FF01-650/60-25	M	Mirror	SP(i)	Semrock F75-750
CM	Cold mirror	obj.	Objective	T	Telescope
DM	Dichroic mirror	PBS	Polarizing beam splitter	TL	Tube lens
fluo.	Fluorescence	PH	Pinhole	VPP	Vortex phase plate

Supplementary Figure S1. Overview of the experimental set-up. The excitation light was provided by a supercontinuum source, and the STED wavelength was 775 nm. Both were operated at a repetition rate of 38.956 MHz. The excitation wavelength was selected by AOTF1 and AOTF3. Beam scanning was achieved by the quad scanner (QS). The fluorescence light was split by various commercial filters (LP(a,c,f,g), BP(j), SP(i)) and collected by four APDs (APD1 – APD4) after passing through a 100 µm pinhole (PH).

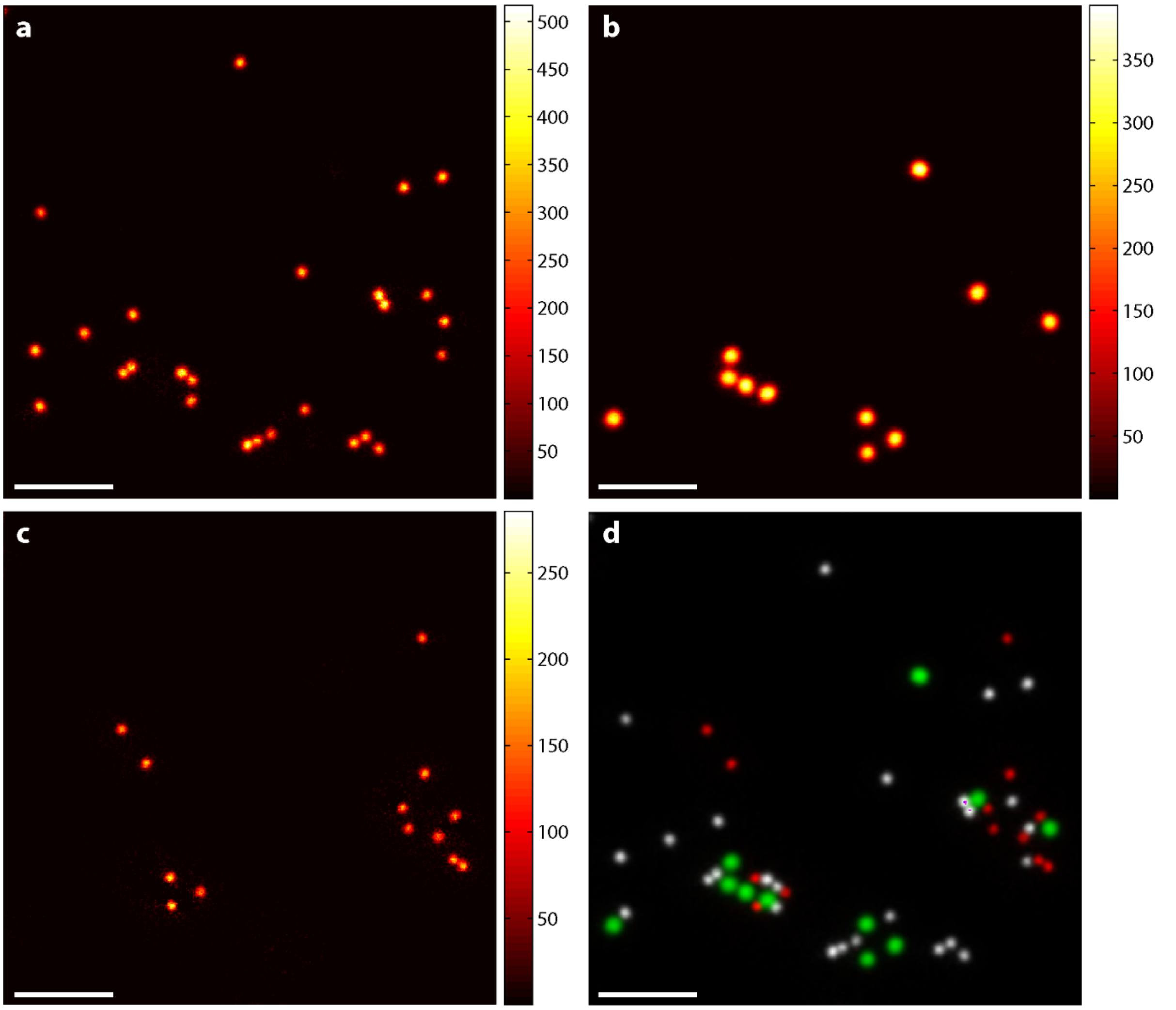

Supplementary Figure S2. Unmixed STED data of a three-colour fluorescent bead sample. The sample consisted of 100 nm crimson (**a**), 200 nm dark red (**b**) and 100 nm far red (**c**) beads. (**d**) overlay of the individual unmixed channels. grey: crimson, green: dark red, red: far red beads. Excitation wavelength was 656 nm (5 µW) and STED wavelength was 775 nm (200 mW). For more details see[1]. Data is counts registered (colourmaps indicated). Scale bars: 1 µm.

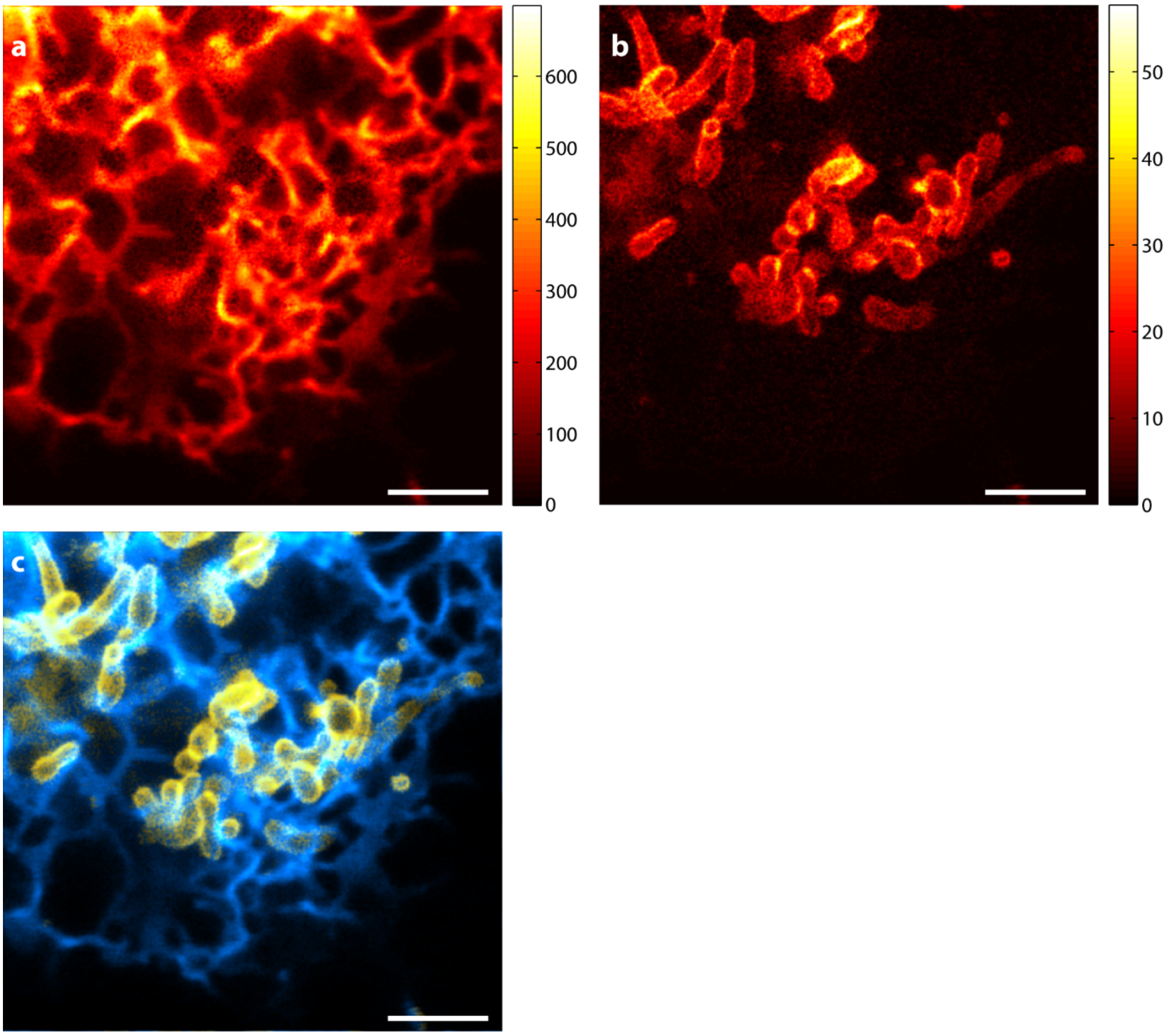

Supplementary Figure S3. Unmixed STED data of a two-colour live-cell sample for calibration. HeLa cells were stained with 580CP-Halo-Sec61β (ER) and 620CP-Snap-OMP25 (outer mitochondrial membrane). (**a**,**b**) unmixed STED data for 580CP (b) and 620CP (b). (**c**) overlay of (a) and (b). Blue: 580CP – ER, yellow: 620CP – mitochondria. Excitation was at 612 nm with 5 µW and a STED power of around 60 mW at 775 nm. Scale bars: 2 µm.

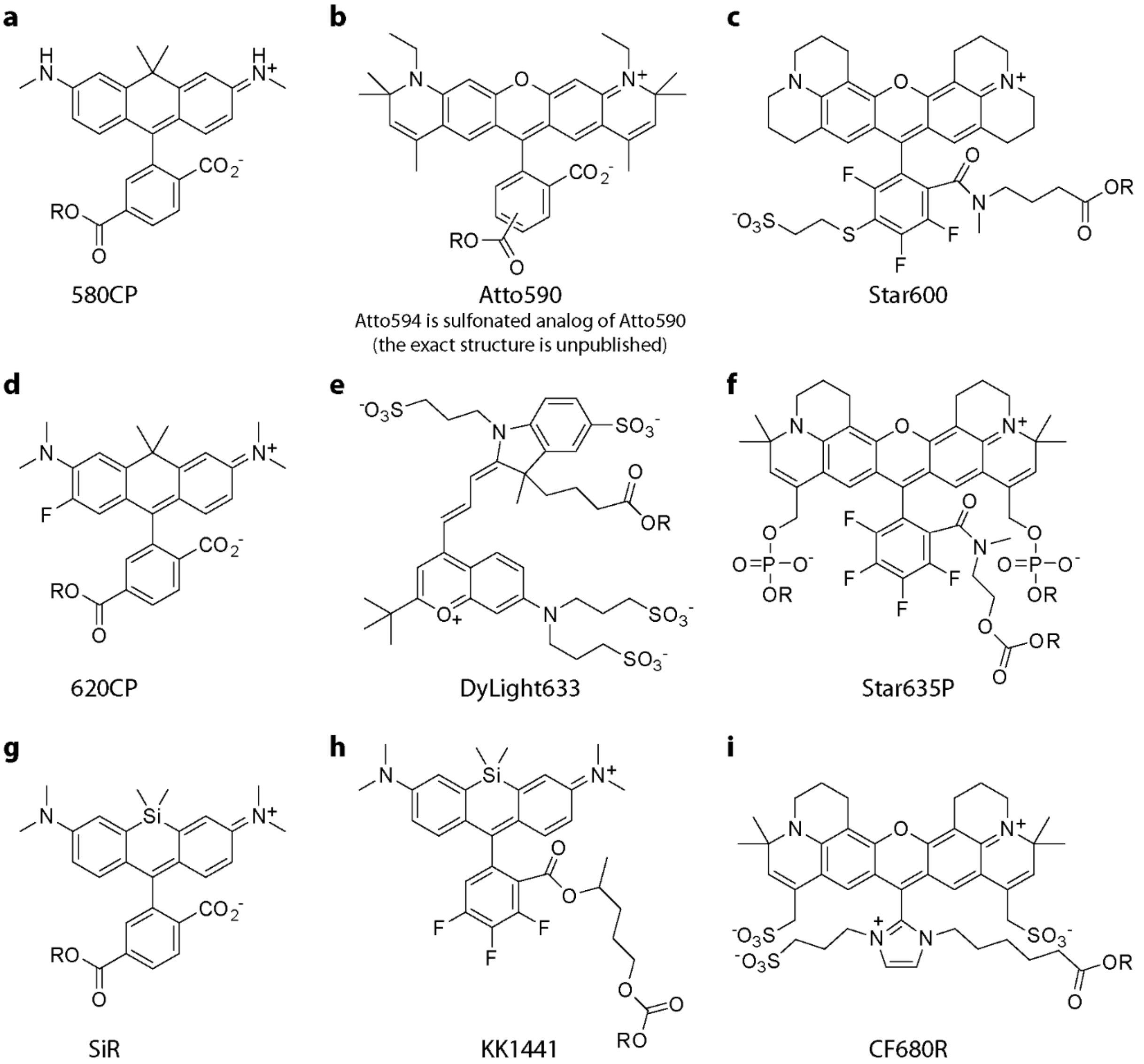

Supplementary Figure S4. Structures of the dyes used in the present study (R = H or N-succinimidyl). (**a**) 580CP, (**b**) Atto590, (**c**) Abberior Star600, (**d**) 620CP, (**e**) DyLight633, (**f**) Abberior Star635P, (**g**) SiR, (**h**) KK1441, (**i**) CF680R.

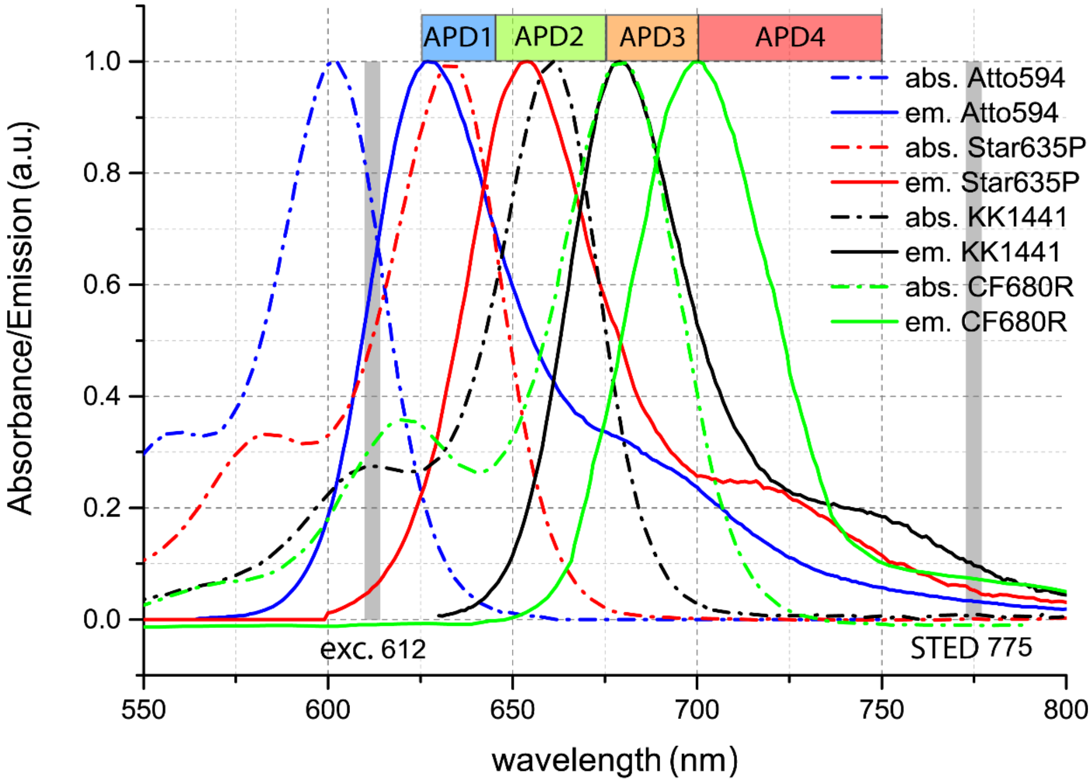

Supplementary Figure S5. Absorption and emission spectra of dyes used in fixed-cell demonstrations of hyperSTED.

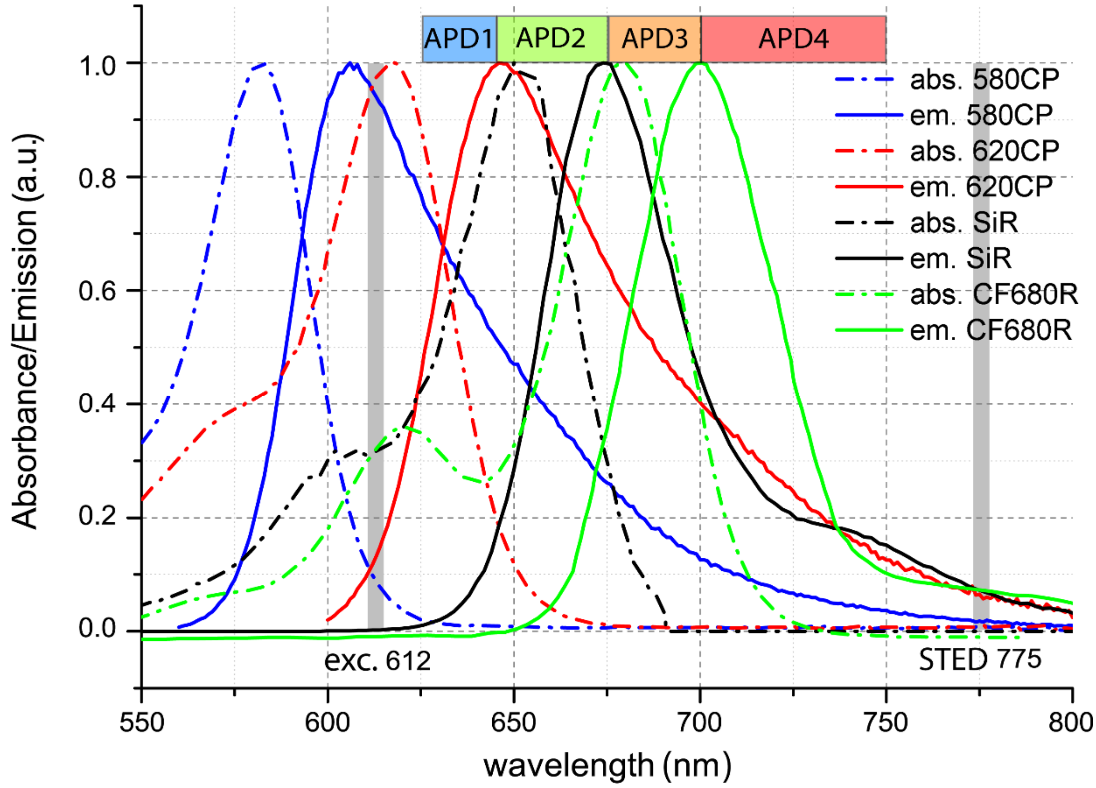

Supplementary Figure S6. Absorption and emission spectra of dyes used in live-cell demonstrations of hyperSTED.

Based on the absorption and emission spectra as well as the detection windows indicated in Supplementary Figs. S5 and S6, bleedthrough can be rationalized by integration. Note however that the spectra may be subject to changes due to coupling to an antibody or embedding in a mounting medium; the spectra shown here represent the dyes in solution as specified by the manufacturers. For the percentage distribution of a certain dye in a specific detection channel see Supplementary Tables S6-S9, where the transfer matrices $\boldsymbol{A}$ for the respective samples are stated.

Supplementary Table S1. Minima and maxima of the adjusted colourmaps of the four-colour fixed-cell sample of Fig. 4 (counts).

	STED		confocal		STED zoom		confocal zoom	
Dye	min	max	min	max	min	max	min	max
Atto594	4.5	165	5.5	69	5	53	3.3	32
Star635P	1	86	8	121	3	65	4	64
KK1441	1.4	160	25	285	5.5	176	15	366
CF680R	6.5	89	18	193	10	102	11	187

Note that the maximum of Atto594 is higher in STED than in confocal imaging, in contrast to the other dyes. This is due to the seven-fold longer acquisition time during STED compared to confocal imaging and the fact that Atto594, being the bluest of the four dyes, has the lowest STED switch-off efficiency, which leaves a larger number of signaling on-state dyes per scanning step.

Supplementary Table S2. Minima and maxima of the adjusted colourmaps of the three-colour live-cell sample of Fig. 5d, f – j (counts).

	Full frame1 (**d**)		Zoom frame1 (**f**)		Zoom frame2 (**g**)		Zoom framz3 (**h**)		Zoom frame4 (**i**)		Zoom frame5 (**j**)	
Dye	min	max	min	max	min	max	min	max	min	max	min	max
580CP	2	42	2	29	2	24	2	19	2	18	2	18
SiR	0	368	0	252	1	253	1	229	1	231	1	213
CF680R	9	47	6	67	6	20	6	55	6	55	6	53

Supplementary Table S3. Minima and maxima of the adjusted colourmaps of the four-colour live-cell sample of Fig. 6e, h – k (counts).

	Full frame1 (**e**)		Zoom frame1 (**h**)		Zoom frame2 (**i**)		Zoom frame3 (**j**)		Zoom frame4 (**k**)	
Dye	min	max	min	max	min	max	min	max	min	max
580CP	1	638	1	638	2	369	0	596	1	578
620CP	69	198	69	198	63	229	64	239	61	204
SiR	3	1068	5	845	4	845	3	845	4	847
CF680R	5	70	5	70	5	70	5	70	5	70

Supplementary Table S4. Residual crosstalk after unmixing by non-negative matrix factorization (NNMF) for a three-colour bead sample. Percentage crosstalk in % determined in a three-colour fluorescent bead sample with 100 nm crimson, 200 nm dark red and 100 nm far red beads. Only pixels above 5 counts were considered for all bead species. The table needs to be read line wise. The bead species whose signal bleeds through is listed left (e.g. read as: 2% of far red beads is bleeding into the unmixed dark red bead channel).

bead/channel	crimson	dark red	far red
crimson	100	1	1
dark red	0	100	0
far red	2	2	100

Supplementary Table S5. Residual crosstalk after unmixing by non-negative matrix factorization (NNMF) for a two-colour live-cell sample. Percentage crosstalk in % determined in a two-colour live-cell sample with 580CP-Halo and 620CP-Snap. Only pixels above 0.1 and 0.5 counts were considered for 580CP and 620CP, respectively. The table needs to be read line wise. The dye whose signal bleeds through is listed left (e.g. read as: 4% of 580CP is bleeding into the unmixed 620CP channel).

bead/channel	580CP	620CP
580CP	100	4
620CP	12	100

Supplementary Table S6. Transfer matrix *A* as used for unmixing the four-colour fixed-cell data of Figs. 3 and 4. Percentage distribution of Atto594, Star635P, KK1441 and CF680R in the different detection channels (APD1 – 4). Columns are normalized to 1.

channel/dye	Atto594	Star635P	KK1441	CF680R
APD1	0.33	0.14	0.01	0.01
APD2	0.45	0.55	0.34	0.12
APD3	0.13	0.19	0.40	0.40
APD4	0.09	0.12	0.25	0.47

Supplementary Table S7. Transfer matrix *A* as used for unmixing the three-colour live-cell data of Fig. 5. Percentage distribution of 580CP, SiR and CF680R in the different detection channels (APD1 – 4). Columns are normalized to 1.

channel/dye	580CP	SiR	CF680R
APD1	0.25	0.02	0.01
APD2	0.55	0.18	0.05
APD3	0.13	0.50	0.49
APD4	0.07	0.30	0.45

Supplementary Table S8. Transfer matrix *A* as used for unmixing the four-colour live-cell data of Fig. 6. Percentage distribution of 580CP, 620CP, SiR and CF680R in the different detection channels (APD1 – 4). Columns are normalized to 1.

channel/dye	580CP	620CP	SiR	CF680R
APD1	0.29	0.20	0.05	0.01
APD2	0.48	0.47	0.53	0.12
APD3	0.14	0.20	0.29	0.40
APD4	0.09	0.13	0.13	0.47

Supplementary Table S9. Transfer matrix *A* as used for unmixing the two-colour live-cell data of Fig. S3. Percentage distribution of 580CP, 620CP, SiR and CF680R in the different detection channels (APD1 – 4). Columns are normalized to 1.

channel/dye	580CP	620CP
APD1	0.29	0.20
APD2	0.48	0.47
APD3	0.14	0.20
APD4	0.09	0.13

Supplementary Note 1. General information and synthesis of 580CP-tubulin, 620CP-Snap and SiR-Halo

NMR spectra. These were recorded at 25 °C with Agilent 400-MR spectrometer at 400.06 MHz (^{1}H), 376.40 MHz (^{19}F) and 100.60 MHz (^{13}C) and are reported in ppm. ^{1}H spectra are referenced to tetramethylsilane (δ = 0 ppm) using the signals of the residual protons of CHD_2OD (3.31 ppm) in CD_3OD; ^{13}C spectra are referenced to tetramethylsilane (δ = 0 ppm) using the signal of CD_3OD (49.00 ppm). Multiplicities of signals are described as follows: s = singlet, d = doublet, dd = doublet of doublets; br = broad signal. Coupling constants (*J*) are given in Hz.

Mass spectra. ESI-MS were recorded on a Varian 500-MS spectrometer (Agilent). ESI-HRMS were recorded on a MICROTOF spectrometer (Bruker) equipped with ESI ion source (Apollo) and direct injector with LC autosampler Agilent RR 1200.

Liquid chromatography. HPLC was performed with Knauer Smartline liquid chromatography system: two pumps (1000), with an UV-detector 2500 with the column thermostat 4000, the mixing chamber and the injection valve with 20 and 100 µL loop for the analytical and preparative columns, respectively; 6-port-3-channel switching valve. Preparative column: Eurospher 100 C18, 5 µm, 250×8 mm, solvent A: acetonitrile, solvent B: H_2O + 0.05% v/v TFA, temperature 25 °C; analytical columns and gradients are indicated in the conditions of the run. Analytical TLC was performed on Merck Millipore ready-to-use plates with silica gel 60 (F_{254}). Preparative TLC was performed on precoated thin-layer plates with silica gel for high performance TLC (HPTLC Silica gel 60 F_{254} 10×10 cm, with concentrating zone 10 x 2.5 cm), purchased from Merck Millipore (Darmstadt, Germany; Cat. No. 113727).

580CP-docetaxel (tubulin)

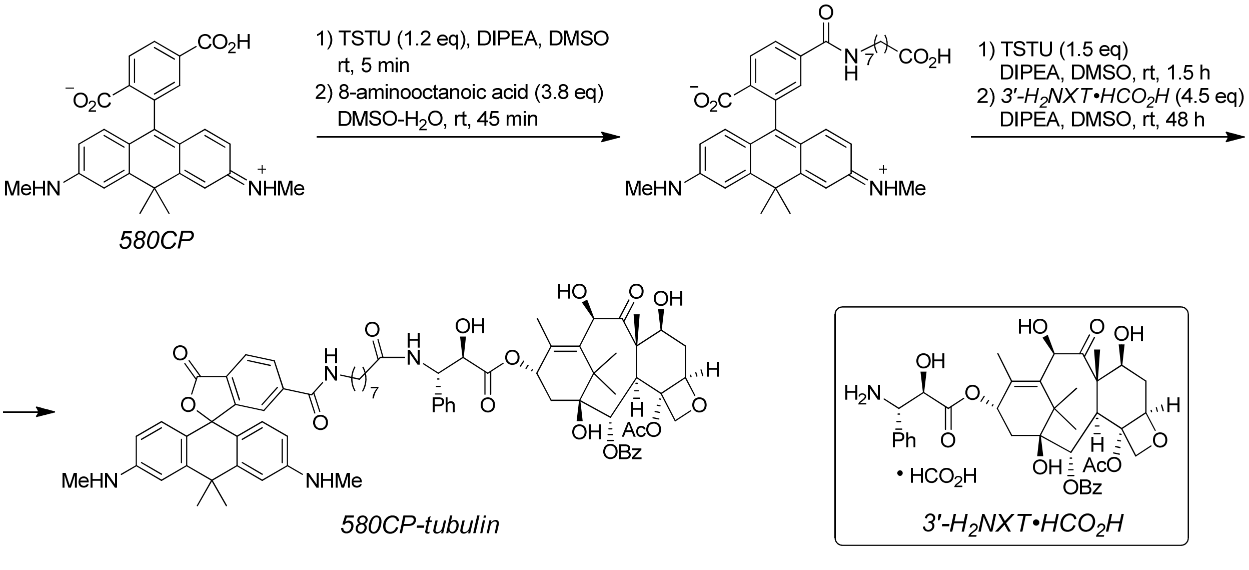

Dye *580CP* (2 mg, 4.6 µmol)[2] and *N,N*-diisopropylethylamine (DIPEA; 21 µL, 120 µmol) were dissolved in DMSO (400 µL). TSTU (*N,N,N',N'*-tetramethyl-*O*-(*N*-succinimidyl)uronium tetrafluoroborate; 1.7 mg, 5.5 µmol, 1.2 eq) was then added and the reaction mixture was stirred for 5 min. 8-Aminooctanoic acid (2.8 mg, 17.5 µmol, 3.8 eq) was then added, and the mixture was sonicated for 15 min at room temperature (rt), followed by addition of water (20 µL) and stirring at rt for further 30 min. The reaction was then quenched by addition of acetic acid (8.3 µL, 30 eq) and the reaction mixture was freeze-dried. The product was isolated by prep. HPLC to give 1.5 mg (56%) of the intermediate acid which was used directly in the next step.

The entire amount of the intermediate (1.5 mg, 2.6 µmol) and *N,N*-diisopropylethylamine (DIPEA; 3.6 µL, 21 µmol) were dissolved under argon atmosphere in dry DMSO (400 µL), and TSTU (10 µL of 3.6 mg/30 µL DMSO stock solution, 4.5 µmol, 1.5 eq) was added. The reaction mixture was stirred at rt for 1.5 h, and upon complete conversion into the NHS ester (HPLC control) another portion of *N,N*-diisopropylethylamine (DIPEA; 3.6 µL, 21 µmol) was added followed by *3'-H₂NXT·HCO₂H* ("3'-aminodocetaxel formate" [3]; 2.9 mg, 4.0 µmol, 1.5 eq). The mixture was stirred at rt, and two additional portions of *3'-H₂NXT·HCO₂H* (2.9 mg, 4.0 µmol, 1.5 eq) were added at 6 h and 20 h from the beginning of the reaction due to incomplete conversion (HPLC control). After 48 h total reaction time, the mixture was freeze-dried and the product (*580CP-tubulin*) was isolated by prep. HPLC. Yield 300 µL of 1.5 mM solution in DMSO (17%), measured by UV-Vis spectroscopy in PBS 7.4 + 0.1% SDS[3].

ESI-MS, positive mode: *m/z* (rel. int., %) = 1259.6 (100) $[M+H]^+$.

HR-MS (ESI, positive mode): 1259.5798 $[M+H]^+$ (found), 1259.5799 (calculated for $C_{72}H_{83}N_4O_{16}$, $[M+H]^+$).

HPLC (Kinetex 2.6 µm C18 100, 4.6 × 75 mm, 1.2 mL/min, gradient A:B = 20:80 → 100:0 over 10 min; A – acetonitrile, B – 0.05% v/v TFA in water, detection at 600 nm): t = 5.4 min.

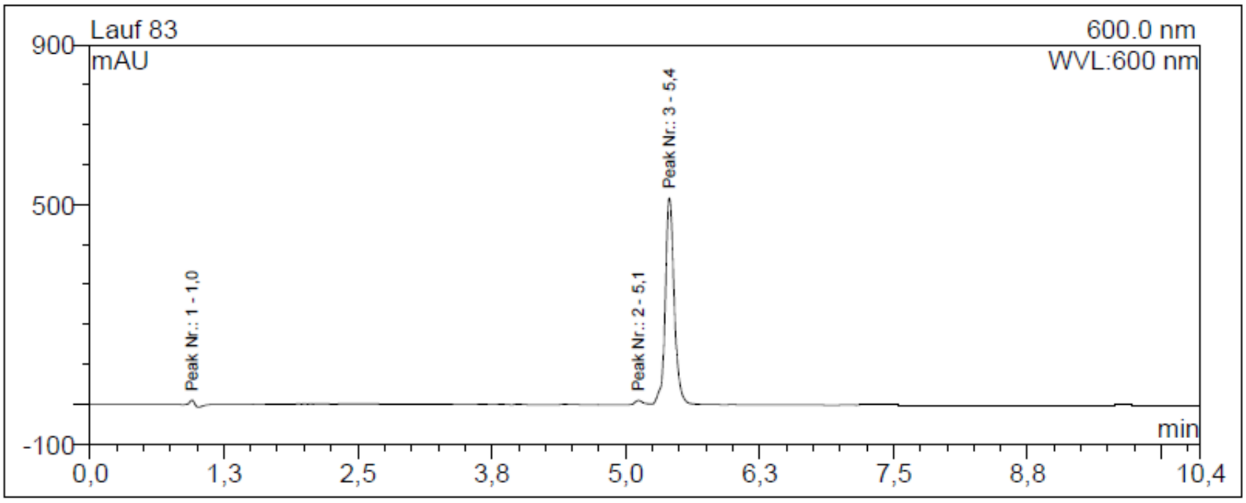

620CP-SNAP

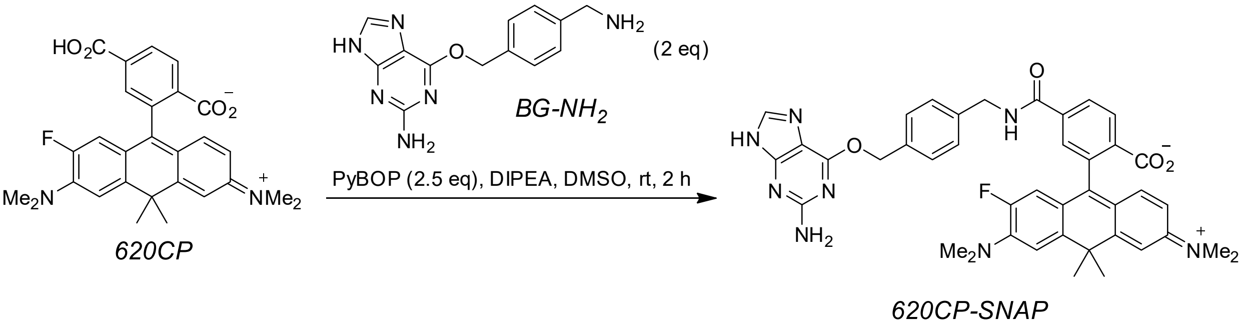

Dye *620CP* (2.4 mg, 5 µmol)[2], BG-NH₂ (6-(4-Aminomethyl)benzyloxy-7*H*-purin-2-amine; 2.7 mg, 10 µmol, 2 equiv) and *N,N*-diisopropylethylamine (DIPEA; 13 µL, 74.8 µmol) were

dissolved in DMSO (100 µL). PyBOP (33 µL of 20 mg/100 µL stock solution in DMSO, 12.5 µmol, 2.5 equiv) was then added, and the mixture was stirred at rt for 2 h. The reaction mixture was poured into water (20 mL), brine (5 mL) was added and the mixture was extracted with ethyl acetate (3 × 15 mL). The combined extracts were dried over Na_2SO_4, filtered and evaporated. The product was isolated by preparative TLC (silica, 10% methanol – CH_2Cl_2) and freeze-dried from aqueous 1,4-dioxane to give 3.0 mg (83%) of *620CP-SNAP* as light blue fluffy solid.

ESI-MS, positive mode: *m/z* (rel. int., %) = 727.3 (100) $[M+H]^+$.

HR-MS (ESI, positive mode): 727.3139 $[M+H]^+$ (found), 727.3151 (calculated for $C_{41}H_{40}N_8O_4F$, $[M+H]^+$).

^{1}H NMR (400 MHz, CD_3OD): δ 8.11 – 8.04 (m, 2H), 7.83 (br.s, 1H), 7.48 – 7.42 (m, 3H), 7.29 (d, J = 8.0 Hz, 2H), 7.18 (d, J = 8.9 Hz, 1H), 6.95 (d, J = 2.6 Hz, 1H), 6.62 (dd, J = 8.9, 2.6 Hz, 1H), 6.54 (d, J = 8.9 Hz, 1H), 6.29 (d, $^3J_{H\text{-}F}$ = 14.0 Hz, 1H), 5.50 (s, 2H), 4.49 (s, 2H), 2.98 (s, 6H), 2.89 (s, 6H), 1.86 (s, 3H), 1.75 (s, 3H).

^{13}C NMR (101 MHz, CD_3OD): δ 129.6 (+), 129.44 (+), 129.43 (+), 128.5 (+), 126.0 (+), 123.5 (+), 117.0 (+), 114.9 (+), 114.7 (+), 113.1 (+), 110.4 (+), 68.4 (–), 49.0 (+), 44.2 (–), 42.6 (+), 40.4 (–), 35.2 (+), 33.6 (+) (indirect detection from HSQC experiment; only H-coupled carbons are resolved).

HPLC (Kinetex Eurospher II 5 µm 100-5 C18, 4 × 100 mm, 1.2 mL/min, gradient A:B = 20:80 → 100:0 over 15 min; A – 0.1% v/v TFA in acetonitrile, B – 0.1% v/v TFA in water, detection at 620 nm and 254 nm): t = 5.5 min.

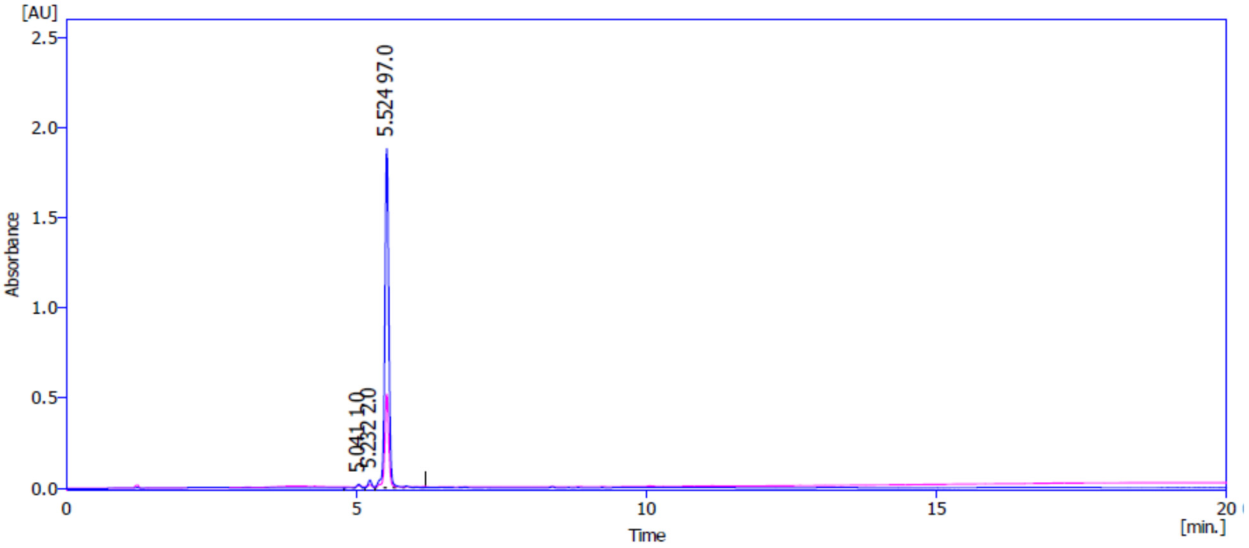

SiR-Halo

The dye *SiR* and its tagged derivative SiR-Halo were prepared according to the reported procedure[4].

Supplementary Note 2. Generation of Tat-EGFP-CF680R. EGFP with an N-terminal Tat peptide (YGRKKRRQRRREF) was expressed from the vector pGEX-6P-1 in *E. coli* SURE cells. Cells were induced with 1 mM IPTG over night at 30 °C. The protein was purified using a GST SpinTrap column (GE Healthcare, Munich, DE) according to the instructions of the manufacturer. The GST-tag was removed by cleavage with PreScission Protease (GE Healthcare, Munich, DE) and repeated application to a GST SpinTrap column. Tat-EGFP was subsequently dialyzed extensively against PBS. The protein was labeled with CF680R NHS ester (Biotium Inc., Hayward, US) according to the manufacturer's instructions, resulting in a degree of labeling of 1.5. Free dye was removed by repeated dialysis against PBS. The final protein concentration was 120 μM.

Supplementary references

1. Winter, F. R. Multicolour STED nanoscopy with hyperspectral detection, Ruperto-Carola University of Heidelberg (2016).
2. Butkevich, A. N. *et al.* Fluorescent Rhodamines and Fluorogenic Carbopyronines for Super-Resolution STED Microscopy in Living Cells. *Angew. Chem. Int. Ed.* **55**, 3290-3294 (2016).
3. Lukinavicius, G. *et al.* Fluorogenic probes for live-cell imaging of the cytoskeleton. *Nat. Meth.* **11**, 731-733 (2014).
4. Lukinavičius, G. *et al.* A near-infrared fluorophore for live-cell super-resolution microscopy of cellular proteins. *Nat. Chem.* **5**, 132-139, (2013).
